# Reply: Calcium, dairy, and prostate cancer

**DOI:** 10.1038/sj.bjc.6603681

**Published:** 2007-03-06

**Authors:** I-M Lee, H D Sesso

**Affiliations:** 1 Harvard University Boston, Massachusetts, USA

**Sir**,

We agree with Dr. Heaney that it is unclear whether there is any association between intakes of dairy products and/or calcium and prostate cancer risk. We had conducted our study, investigating the possibility that higher intakes of dairy products and/or calcium may be associated with increased risk, because calcium suppresses 1,25-dihydroxyvitamin D [1,25(OH)_2_D], which inhibits the proliferation of prostate cancer cells and promotes their differentiation (Koh *et al*, 2006).

We respectfully disagree with Dr. Heaney's statement that serum 1,25(OH)_2_D is unrelated to prostate cancer risk. The data are presently not clear: some studies have shown strong (Corder *et al*, 1993) or weak inverse associations (Gann *et al*, 1996), while others have observed U-shaped (i.e., increased prostate cancer risk with both low and high levels of 1,25(OH)_2_D) (Tuohimaa *et al*, 2004) or null associations (Nomura *et al*, 1998). Additionally, two other studies have reported inverse associations between serum 25-hydroxyvitamin D [25(OH)D] (Ahonen *et al*, 2000) or predicted serum 25(OH)D levels and prostate cancer risk (Giovannucci *et al*, 2006).

Thus, it is biologically plausible – although not proven – for high intakes of dairy products and/or calcium to be related to increased prostate cancer risk. While our study and others (Severi *et al*, 2006) did not observe any association, other large, well-conducted studies have reported that high intakes are indeed predictive of increased prostate cancer risk (Giovannucci *et al*, 1998; Chan *et al*, 2001). Further studies are needed to clarify the association.

## References

[bib1] Ahonen MH, Tenkanen L, Teppo L, Hakama M, Tuohimaa P (2000) Prostate cancer risk and prediagnostic serum 25-hydroxyvitamin D levels (Finland). Cancer Causes Control 11: 847–852 11075874 10.1023/a:1008923802001

[bib2] Chan JM, Stampfer MJ, Ma J, Gann PH, Gaziano JM, Giovannucci EL (2001) Dairy products, calcium, and prostate cancer risk in the Physicians' Health Study. Am J Clin Nutr 74: 549–554 11566656 10.1093/ajcn/74.4.549

[bib3] Corder EH, Guess HA, Hulka BS, Friedman GD, Sadler M, Vollmer RT, Lobaugh B, Drezner MK, Vogelman JH, Orentreich N (1993) Vitamin D and prostate cancer: a prediagnostic study with stored sera. Cancer Epidemiol Biomarkers Prev 2: 467–472 8220092

[bib4] Gann PH, Ma J, Hennekens CH, Hollis BW, Haddad JG, Stampfer MJ (1996) Circulating vitamin D metabolites in relation to subsequent development of prostate cancer. Cancer Epidemiol Biomarkers Prev 5: 121–126 8850273

[bib5] Giovannucci E, Liu Y, Rimm EB, Hollis BW, Fuchs CS, Stampfer MJ, Willett WC (2006) Prospective study of predictors of vitamin D status and cancer incidence and mortality in men. J Natl Cancer Inst 98: 451–459 16595781 10.1093/jnci/djj101

[bib6] Giovannucci E, Rimm EB, Wolk A, Ascherio A, Stampfer MJ, Colditz GA, Willett WC (1998) Calcium and fructose intake in relation to risk of prostate cancer. Cancer Res 58: 442–447 9458087

[bib7] Koh KA, Sesso HD, Paffenbarger Jr RS, Lee IM (2006) Dairy products, calcium and prostate cancer risk. Br J Cancer 95: 1582–1585 17106437 10.1038/sj.bjc.6603475 PMC2360740

[bib8] Nomura AM, Stemmermann GN, Lee J, Kolonel LN, Chen TC, Turner A, Holick MF (1998) Serum vitamin D metabolite levels and the subsequent development of prostate cancer (Hawaii, United States). Cancer Causes Control 9: 425–432 9794175 10.1023/a:1008875819232

[bib9] Severi G, English D, Hopper J, GG G (2006) Re: prospective studies of dairy product and calcium intakes and prostate cancer risk: a meta-analysis. J Natl Cancer Inst 98: 794–795 16757704 10.1093/jnci/djj215

[bib10] Tuohimaa P, Tenkanen L, Ahonen M, Lumme S, Jellum E, Hallmans G, Stattin P, Harvei S, Hakulinen T, Luostarinen T, Dillner J, Lehtinen M, Hakama M (2004) Both high and low levels of blood vitamin D are associated with a higher prostate cancer risk: a longitudinal, nested case–control study in the Nordic countries. Int J Cancer 108: 104–108 14618623 10.1002/ijc.11375

